# CXCL10 Is Critical for the Generation of Protective CD8 T Cell Response Induced by Antigen Pulsed CpG-ODN Activated Dendritic Cells

**DOI:** 10.1371/journal.pone.0048727

**Published:** 2012-11-07

**Authors:** Saikat Majumder, Surajit Bhattacharjee, Bidisha Paul Chowdhury, Subrata Majumdar

**Affiliations:** Division of Molecular Medicine, Bose Institute, P1/12, C.I.T. Scheme VII-M, Kolkata, India; Federal University of São Paulo, Brazil

## Abstract

The visceral form of leishmaniasis is the most severe form of the disease and of particular concern due to the emerging problem of HIV/visceral leishmaniasis (VL) co-infection in the tropics. Till date miltefosine, amphotericin B and pentavalent antimony compounds remain the main treatment regimens for leishmaniasis. However, because of severe side effects, there is an urgent need for alternative improved therapies to combat this dreaded disease. In the present study, we have used the murine model of leishmaniasis to evaluate the potential role played by soluble leishmanial antigen (SLA) pulsed-CpG-ODN stimulated dendritic cells (SLA-CpG-DCs) in restricting the intracellular leishmanial growth. We found that mice vaccinated with a single dose of SLA-pulsed DC stimulated by CpG-ODN were protected against a subsequent leishmanial challenge and had a dramatic reduction in parasite burden along with the generation of parasite specific cytotoxic T lymphocytes. Moreover, we demonstrate that the induction of protective immunity conferred by SLA-CpG-DCs depends entirely on the CXC chemokine IFN-γ-inducible protein 10 (CXCL10; IP-10). CXCL10 is directly involved in the generation of a parasite specific CD8^+^ T cell-mediated immune response. We observed significant reduction of CD8^+^ T cells in mice depleted of CXCL10 suggesting a direct role of CXCL10 in the generation of CD8^+^ T cells in SLA-CpG-DCs vaccinated mice. CXCL10 also contributed towards the generation of perforin and granzyme B, two important cytolytic mediators of CD8^+^ T cells, following SLA-CpG-DCs vaccination. Together, these findings strongly demonstrate that CXCL10 is critical for rendering a protective cellular immunity during SLA-CpG-DC vaccination that confers protection against *Leishmania donovani* infection.

## Introduction

Among the various antigen presenting cells (APCs) of the immune system, dendritic cells (DC) are critical for the adaptive immune response as they acts as conduits between the innate and adaptive arms of the immune system [1]. Exogenous administrations of antigen-loaded DCs have shown promising results in the treatment of visceral leishmaniasis [2], [3], [4]. However, the roles of toll like receptor ligand-activated DCs in the containment of *L.donovani* infection needs further elucidation. Toll like Receptors are a type of germ-line, pattern recognition receptors that recognize pathogen-associated molecular patterns (PAMPs) [5]. Therefore, TLR activation on DCs helps in mounting a more prominent and directed T cell response [6] and superior killing of the invading pathogen.

CpG oligodeoxynucleotides (ODN) containing unmethylated CpG motifs serve as TLR9 ligands can stimulate DC activation and maturation to professional APCs [7], [8]. CpG-ODN enhances humoral responses, driving them toward IgG2a isotypes, a Th1 type indicator [9], and induces development of enhanced cytotoxic T lymphocyte (CTL) activity [10] by cross-presenting extracellular antigens to CTLs which destroy the antigen-specific pathogens in vivo [11]. TLR ligand–stimulation of DCs causes the production of chemokines, creating gradients that attract naive CTLs and increase the possibility of their encounter with the DCs [12]. Chemokines (8–10 kDa) are an integral part of the host defence against pathogens and have been subdivided into 2 major subfamilies, CC and CXC chemokines [13], [14]. Amongst the latter, CXCL10 is produced by dendritic cells and various others activated human immune cells following CpG-ODN stimulation [15], [16].

CXCL10, with known antitumor, antiviral, and antifungal activities [17]–[19], is essential for the generation of protective CD8^+^ T cell responses [20]. An early and strong induction of CXCL10 accompanies healing in *L.donovani* infected B6 mice, while treatment with exogenous CXCL10 renders protection against *L. donovani* infection, highlighting its relevance in aborting the leishmanial pathogenesis [21], [22]. CXCL10 binds to CXCR3, a seven-transmembrane G protein-coupled receptor expressed on T cells that induces chemotaxis [23]. CXCR3 is critical for T cell activation [24]. CXCR3^−/−^ mice are highly susceptible to infection due to impaired perforin and granzyme B expression by CTLs associated with reduced cytotoxic activity [25]. Collectively, these observations stimulated us to determine the role of CXCL10 in TLR ligand-activated DC based vaccinations, as it can promote the activation of parasite specific CD8^+^ CTLs *in vivo*.

This study demonstrates for the first time, that the induction of an anti-leishmanial CD8^+^ T cell response, following SLA pulsed-CpG-ODN stimulated DC based vaccination is entirely dependent on the production of CXC chemokine CXCL10. Studies in a murine model of visceral leishmaniasis showed that CXCL10 plays a key role in generating parasite-specific-CTLs following SLA-CpG-DC vaccination along with effective killing of the parasite. This study might provide crucial cues in understanding the immunostimulatory role of SLA-CpG-DCs in rendering protection against experimental VL.

## Materials and Methods

### Ethics Statement

This study was carried out in strict accordance with the recommendations in the Guide for the Care and Use of Laboratory Animals of the National Institutes of Health. All experimental animal protocols received prior approval from the Institutional Animal Ethical Committee (Bose Institute, Registration Number: 95/99/CPCSEA).

### Animals, Parasites and Reagents

BALB/c mice were purchased from the National Center for Laboratory Animal Sciences, India. For each experiment 8–10 mice (4–6 weeks old) were used, regardless of sex. *L. donovani* strain AG-83 (MHOM/IN/1983/AG-83) was maintained *in vitro* in Medium 199 (Sigma) containing 10% fetal calf serum (FCS; Gibco BRL). Experiments were performed with stationary phase promastigotes. The CpG oligodeoxynucleotide 1826 (5′-TCCATGACGTTCCTGACGTT-3′) and the control ODN (non-CpG ODN, 5′-TCCATGAGCTTCCTGAGCTT-3′) were obtained from Invivogen. TLR9 siRNA was procured from Santacruz Biotechnology. CXCL10 depleting antibody was obtained from R&D systems. CD8 depleting antibody was obtained from Taconic Laboratories, Germantown, NY; clone TIB210.

### Preparation of Dendritic Cells

Bone marrow-derived DCs from BALB/c mice were generated described previously [26]. Nonadherent cells were collected, and 1×10^6^ cells were placed in plates containing 1 ml of complete medium with GM-CSF (150 U/ml; R&D systems) and IL-4 (75 U/ml; R&D systems). Half of the medium was replaced on day 3, 5 and 7 and fresh medium containing GM-CSF and IL-4 was added. On day 8 of culture, most cells had acquired typical dendritic morphology. These cells were used as the source of DCs in subsequent experiments.

### DC Vaccination

For DC-based vaccination four sets were used. DCs were pulsed with either SLA for 18 hours or CpG-ODNs for 18 hours or with both SLA and ODNs (CpG-ODNs or control-ODN) [3]. In case of dual stimulation, CpG-ODN (10 µg/ml) or control-ODN (10 µg/ml) were added to the media for last 6 hours after 12 hours of SLA stimulation. DCs were then washed with PBS thrice and injected i.v. (10^6^ cells in 100 µl of PBS/mouse) into mice through the tail vein. One week later, mice were infected intravenously with 1×10^7^ stationary phase *L. donovani* promastigotes. Mice were sacrificed on days 1, 7, 14, 28, and 56 post infections. Spleen and liver parasitic loads were determined from giemsa-stained impression smears, calculated as the number of parasites per 1000 nucleated cells x organ weight (in mg) and expressed in Leishman Donovan Units (LDU) [27]. For re-infection study mice were infected intravenously with 1×10^7^ stationary phase *L. donovani* promastigotes 4 weeks after the original infection. Mice re-infected were sacrificed at 12- wk of initial infection and organ parasite burden was determined as above.

### *In vivo* Depletion of CXCL10 and CD8^+^ T Cells

For *in vivo* depletion of CXCL10, anti-mouse CXCL10 mAb (R&D Systems) were injected intraperitoneal (i.p.) on day 0 (250 µg) day 2 (100 µg) and day 4 (100 µg) after SLA-CpG-DCs vaccination. These mice were subsequently infected with 1×10^7^ stationary phase *L. donovani* promastigotes after 7 days of initial vaccination. 250 µg of anti- CXCL10 mAb was again injected i.p on days 10, 15, and 24 of initial vaccination. CD8^+^ T cells were depleted by one i.p. injection of CD8 depleting antibody (clone TIB210) (500 µg) 1 day before the vaccination. Depletion efficiencies were assessed at regular intervals with mAbs by flow cytometric analysis.

### Purification of CD8^+^ T Cells

CD8^+^ T cells were purified from splenocytes from differently treated mice by positive selection using magnetic beads (Mouse CD8 T Lymphocyte Enrichment Set; BD Biosciences). The CD8^+^ population purity was routinely confirmed to be around 98%.

### Proliferation Assay

Splenic CD8^+^ T cells (10^6^) were cultured for 4 days in 96-well, round-bottom plates. SLA (10 µg/ml) or ConA (2.5 µg/ml) was added to the culture medium for stimulation for 72 hours. One µCi of [^3^H] thymidine (JONAKI, DAE) was added 18 h before harvest and incorporated radioactivity was measured using a liquid scintillation counter (Tri-Carb 2100TR; Packard Instrument).

### Cytokine and Chemokine ELISA

Culture supernatants were analyzed using a sandwich ELISA kit (Quantikine M; R&D Systems), in accordance with the manufacturer’s instructions. Briefly, 50 µL of Assay Diluent (10% FCS in PBS) was added in ELISA plates coated with capture antibody. The plates were then incubated for 1 hr at RT followed by subsequent washing with wash buffer (0.05% Tween 20 in PBS). Then 100 µL of samples were added in respective plates and were incubated at RT for another 2 hr. After completion of incubation, the wells were aspirated and washed followed by addition of respective antibody conjugate (Detection Ab + SAv-HRP). The plates were then incubated for 2 hr at RT. After washing 100 µL substrate solution was added to each well. The plates were then kept in dark for 30 minutes. Finally 100 µL Stop Solution was added to each well and reading was taken within 30 minutes using a microplate reader set to 450 nm.

### Flow Cytometry Analysis and Abs

For FACS analysis, DCs or CD8^+^ T cells(1×10^6^) were prepared as described elsewhere (22). DCs were stained with fluorescently labelled Abs anti-CD11c-PE, anti-CD80–PE, anti-CD86–PE, and anti-MHCII-PE in separate sets for 60 min at 4°C in dark and washed three times before acquisition. Similarly, purified CD8^+^ T cells were stained with anti-Granzyme B–PE and anti-Perforin-FITC Abs for 60 min at 4°C in dark. Intracellular cytokine staining of purified CD8^+^ T cells with anti-IFN gamma-PE was performed using a Cytofix/Cytoperm-Plus Kit with GolgiPlug (BD Pharmingen) as per the manufacturer’s instructions. Cells were then washed and analyzed using a FACS calibur (BD Biosciences). A minimum of 10,000 events was collected for each sample, and data analysis was performed with the CellQuest software (BD biosciences).

**Figure 1:**
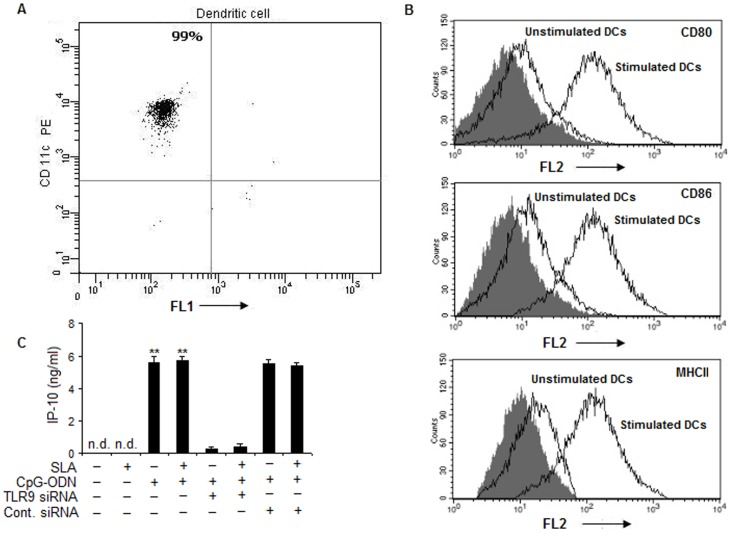
Immunophenotyping of BMDCs. (**A**) Purity of dendritic cells. BALB/c mice bone marrow cells were cultured in the presence of GM-CSF and IL-4 for 7 days. Then these bone marrow-derived DCs (BMDCs) were washed and adhered for 3 h. The adherent cells were stained with CD11c-PE and analyzed on a flow cytometer. (**B**) Purified DCs were stained with CD80-PE, CD86-PE and MHC class II-FITC antibodies to detect their maturation levels following CpG-ODN (10 µg/ml) stimulation. The data show histograms of cell number against fluorescence intensity and are representative of three experiments. Solid areas indicate staining with isotype matched antibodies. Lines show staining of stimulated and un-stimulated DCs with the indicated mAbs. (**C**) DCs (1×10^6^ cells /ml) were stimulated with SLA (10 µg/ml) or CpG-ODN (10 µg/ml) alone or in combinations for 24 hours. In some experimental sets, DCs were transfected with TLR9-specific siRNA or control siRNA. Briefly, DCs (10^6^ cells) were transfected with TLR9 siRNA at a final concentration of 100 nM, using transfection reagent Oligofectamine (Invitrogen, Carlsbad, CA, USA) as per manufacturer’s instructions. The DCs were incubated with the transfection complexes in RPMI without serum for 6 h followed by another 18 h with 10% FBS. Control cultures were transfected using scrambled TLR9 siRNA (100 nM). Cells were then washed, and treated with SLA and CpG-ODN. Cell supernatants were then obtained for detecting CXCL10 levels by ELISA. Data are means ± standard deviations of values from 3 independent experiments conducted in the same way that yielded similar results. **P<.001, compared to unstimulated control. n.d. not detectable.

### Isolation of RNA and RT-PCR

RNA was isolated according to the standard protocol using TRIZOL™ reagent (SIGMA) from purified CD8^+^ T cells isolated from different groups of mice. Isolated total RNA was then reverse transcribed using Revert Aid ™ M-MuLV Reverse Transcriptase (Fermentas). The resulting complementary DNA was used for reverse transcriptase PCR with Perkin Elmer Gen Amp PCR 2400 system. PCR amplification was conducted in a reaction volume of 50 µl with 0.5U of Taq polymerase for 35 cycles (denaturation at 94°C for 30 s, annealing at 58°C for 30 s, and extension at 72°C for 30 s. The PCR-amplified product was subsequently size fractioned on a 1% agarose gel, stained with ethidium bromide, and visualized under UV light. Sequences of the PCR primers used were as follows: Perforin:Forward 5′-CTGAGCGCCTTTTTGAAGTC-3′; Reverse 5′-AAGGTAGCCAATTT- TGCAGC-3′, Granzyme B:Forward*-*5′-CTCTCGAATAAGGA- AGCCCC-3′; Reverse 5′-CTGACCTTGTCTCTGGCCTC-3′and GAPDH: Forward 5′-CAAGGCTGTGGGCAAGGTCA-3′; Reverse 5′-AGGTGGAAGAGTGGGAGTTGCTG3′.

**Figure 2:**
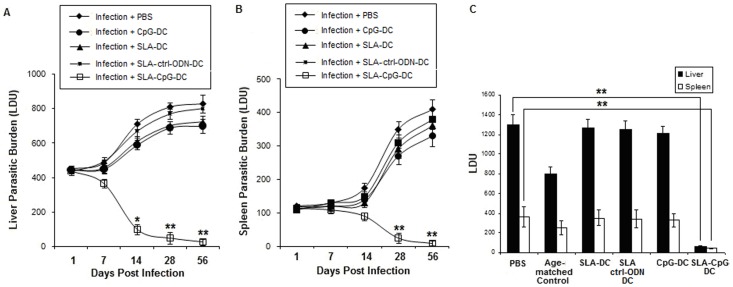
Effect of *in vivo* treatment with SLA-CpG-DCs on the parasite load in liver and spleen of Leishmania donovani–infected BALB/c mice. Mice were either vaccinated with SLA-pulsed, SLA+ CpG-ODN pulsed, SLA+ control-CpG-ODN pulsed, only CpG-ODN-pulsed DCs or either phosphate buffered saline (PBS; control) followed by intravenous infection with 1×10^7^ stationary phase *Leishmania donovani* promastigotes after 7 days. Mice were sacrificed on days 1, 7, 14, 28, and 56 after infection. Levels of parasite burden in liver (A) and spleen (B) samples were determined by stamp-smear method and expressed in Leishman Donovan Units (LDU). Results are from 3 independent experiments and represent the mean values ± standard errors of the means for 5 animals per group per time point. **P<.001 and *P<.005, compared to infected mice. (C) Cured mice after 5 weeks of vaccination (4 weeks of infection) along with age-matched controls were re-infected with similar dose of *Leishmania donovani* and at 12 weeks of primary infection, were sacrificed and liver and spleen parasitic loads were determined by stamp-smear method and expressed as Leishman Donovan Units (LDU). Results are from 3 independent experiments and represent the mean values ± standard errors of the means for 5 animals per group per time point. **P<.001, compared to infected mice.

### Cytotoxicity Assay of CD8^+^ T Cells

To assess the cytotoxicity of antigen-primed CD8^+^ T cells, purified CD8^+^ T cells (2×10^5^) were isolated from SLA-CpG-DCs vaccinated and CXCL10 depleted SLA-CpG-DCs vaccinated *L. donovani* infected mice 28 days following infection and were co-cultured with autologous *L.donovani*-infected macrophages in a 10∶1 ratio. After 4 h, the co-cultured cells were harvested and stained with anti-CD14-FITC antibodies to select the target macrophages. They were also analyzed by propidium iodide staining to detect the killed macrophages in flow cytometer.

**Figure 3:**
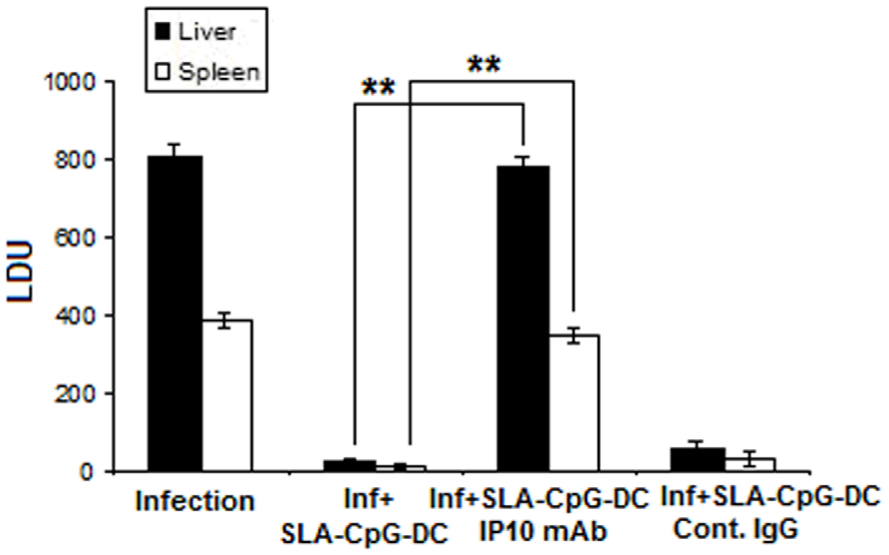
Effect of CXCL10 depletion on protective immunity induced by the SLA-CpG-DCs. For *in vivo* depletion, anti-mouse CXCL10 mAb were injected i.p. on day 0 (250 µg) day 2 (100 µg) and day 4 (100 µg) after SLA-CpG-DCs vaccination. These mice were subsequently infected with 1×10^7^ stationary phase *Leishmania donovani* promastigotes after 7 days of initial vaccination. 250 µg of anti- CXCL10 mAb was again injected i.p on days 10, 15, and 24 of initial vaccination. In one group of similarly vaccinated and infected mice a control IgG Ab was injected. All groups of mice were sacrificed on day 28 after infection. Levels of parasite burden in liver and spleen samples were determined by stamp-smear method and expressed in Leishman Donovan Units (LDU). Results are from 3 independent experiments and represent the mean values ± standard errors of the means for 3 animals per group. **P<.001, compared to SLA-CpG-DC vaccinated infected mice.

### Statistical Analysis

A minimum of five mice were used per group for *in vivo* experiments. The data, represented as mean ±standard deviation (SD), is from one experiment, which was performed at least three times. Student’s t test was employed to assess the significance of the differences between the mean values of control and experimental groups. A P value of 0.005 was considered significant and less than 0.001 was considered highly significant.

**Figure 4:**
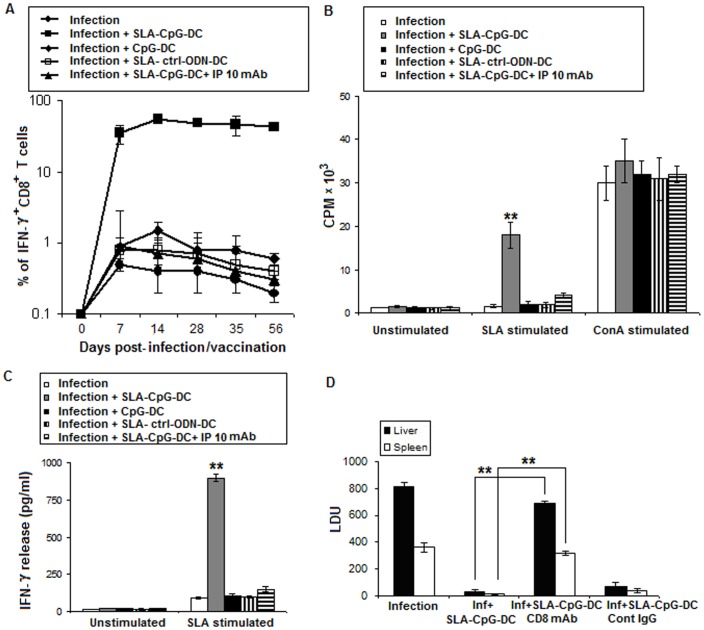
Vaccination with SLA-CpG-DCs induces a CXCL10 mediated CD8^+^ T cell response in parasitized mice. (**A**) Spenocytes (1×10^6^) were isolated from spleen of infected, differently vaccinated and CXCL10 depleted vaccinated mice after 0, 7, 14, 28, 35 and 56 days after infection. Splenocytes were then harvested, stimulated for 4 h with SLA (10 µg/ml), and then stained for IFN-γ and CD8. Then cells were analyzed for expression of CD8 and IFN-γ on a flow cytometer. The values shown (plotted on a log10 scale) reflect the average number of antigen-specific IFN-γ^+^CD8^+^ T cells expressed as a percentage of total CD8^+^ T cells present in the spleens. Results are from 3 independent experiments and represent the mean values ± standard errors of the means for 5 animals per group per time point. (**B**) Increased CD8^+^ T Cell Proliferation in CD8^+^ T cells purified from SLA-CpG-DC vaccinated parasitized mice. CD8^+^ T cells (1×10^6^) were purified from spleens of infected, differently vaccinated mice and CXCL10 depleted vaccinated mice (methods) after 4 weeks post infection and were stimulated in presence of ConA (2.5 µg/ml) or in presence or absence of SLA (10 µg/ml) and after 72 h incubation, proliferation was measured by ^3^H thymidine incorporation. Values and bars represent mean CPM and the standard deviation and are representative of three independent experiments. **P<.001, compared to unstimulated controls. CPM: counts per minute (**C**) IFN-γ production was enhanced in CD8^+^ T cells purified from SLA-CpG-DC vaccinated parasitized mice. CD8^+^ T cells (1×10^6^) were purified from spleens of differently vaccinated mice (methods) after 4 weeks post infection and plated with T-depleted, mitomycin C-treated syngeneic APCs (5×10^5^). The cells were then stimulated in presence or absence of SLA (10 µg/ml) for 72 h. Samples were assayed in triplicate by ELISA. Values and bars represent mean CPM and the standard deviation. The experiments were repeated thrice more with similar results. **P<.001, compared to unstimulated controls. (**D**) Contribution of CD8^+^ T cells to SLA-CpG-DCs mediated vaccination against *Leishmania donovani.* CD8^+^ T cells were depleted by one intraperitoneal (i.p.) injection of CD8 depleting antibody (100 µg) 1 day before the vaccination. In one group of similarly vaccinated and infected mice a control IgG Ab was injected. Mice were subsequently immunized i.v. with SLA-CpG-DCs and were challenged with *L. donovani* promastigotes 7 days later. Control mice were treated with PBS. Mice were sacrificed on day 28 after infection. Levels of parasite burden in liver and spleen samples were determined by stamp-smear method and expressed in Leishman Donovan Units (LDU). Results are from 3 independent experiments and represent the mean values ± standard errors of the means for 3 animals per group per time point. **P<.001 compared to SLA-CpG-treated infected mice.

## Results

### 1. SLA-pulsed DCs Exposed to CpG ODN Cures Established Murine Visceral Leishmaniasis

To establish whether matured antigen-primed DCs could generate a host-protective immune response in *L.donovani donovani*-susceptible BALB/c mice, DCs were generated. The purity of these DCs was found to be above 98% (Figure 1A) and these DCs expressed high amount of MHC class II and co-stimulatory molecules CD80 and CD86 following in vitro stimulation with CpG-ODN (Figure 1B). We also observed that *in vitro* stimulation of DCs with CpG-ODN resulted in the significantly high production of CXCL10, a CXC chemokine. However, pulsing these DCs with both CpG-ODN and SLA did not cause any increase in the secretion of CXCL10 suggesting that SLA has no role in IP- 10 production by these DCs. There was significant reduction of CXCL10 level in TLR 9 silenced condition; thereby highlighting the role of TLR9 in CpG-ODN induced CXCL10 generation (Figure 1C). Besides, the control siRNA did not have any reducing effect on the production of CXCL10 (Figure 1C).

To evaluate the maturation requirements enabling DCs to generate a protective immune response differently pulsed DCs were subsequently injected into BALB/c mice which were challenged with *L. donovani* intravenously 1 week later, and the course of disease was monitored. Our results demonstrated that when DCs were SLA-pulsed in the presence of a prominent Toll-like receptor (TLR) agonist, CpG ODN (SLA-CpG-DCs), they acquired the ability to induce protection in susceptible BALB/c mice against an otherwise lethal infection with *L. donovani*. SLA-CpG-DCs vaccinated mice on day 28 (4 weeks) of infection, showed marked decrease in parasitic burden by about 92% in liver and 89% in spleen (Figure 2A–B) and this protection was seen to persist till 56 days (8 weeks) of infection; 96±2.6% and 97±1.9% reduction in hepatic and splenic parasite burden respectively after 56 days of infection, compared with PBS-treated infected controls (Figure 2A–2B). Mice treated with SLA and control-CpG-ODN-pulsed DCs (SLA-Ctrl-DCs) or only CpG-ODN pulsed DCs (CpG-DCs) showed a course of disease comparable to that in the PBS-treated infected group (Figure 2A–2B).

We observed significant reduction of parasitic burden (92% in liver and 89% in spleen; Figure 2A–B) in mice 5 wks post vaccination. To determine whether these SLA-CpG-DCs vaccinated BALB/c mice which resolved the primary infection were able to resist a second infection, they were re-infected with *L. donovani* intravenously at 5-week post-vaccination (4-week after initial infection) and were sacrificed at 12-week of initial infection and organ parasite burden was determined. Age-matched normal mice were also infected simultaneously and were considered as age-matched controls. We found that, mice vaccinated with SLA-CpG-DCs were more resistant to re-infection with *L. donovani* (Figure 2C) compared to only CpG-DCs or SLA-Ctrl-DCs vaccinated mice.

### 2. Effect of CXCL10 Depletion on Protective Immunity Induced by the SLA-CpG-DCs

The role of CXCL10 was assessed in the induction of a protective immunity induced by the SLA-CpG-DCs. For this purpose, BALB/c mice were vaccinated by SLA-CpG-DCs, challenged 7 days thereafter by *L.donovani*, and analyzed for hepatic and splenic parasitic burden, in the absence of CXCL10. The depletion of CXCL10 by anti- CXCL10 mAbs for the duration of 4 weeks following infection (5 weeks following vaccination) abrogated the protective immunity induced by vaccination with SLA-CpG-DCs (Figure 3). The apparent lack of protection in CpG-DCs vaccinated sets, despite their high production of CXCL10 when stimulated with CpG-ODN in vitro, suggests that indirect signaling via TLR is not adequate to stimulate the expansion of host-protective Th1-response, signifying that the host-protective response was reliant on both CpG-ODN mediated DC stimulation and leishmanial antigen presentation by these DCs.

### 3. Vaccination with SLA-CpG-DCs Induces a CXCL10 Mediated CD8^+^ T Cell Response in Parasitized Mice

CXCL10 plays a critical role in the effector CD8^+^ T cell generation and was necessary for its trafficking [28]. CD8^+^ T cells are primarily responsible for protection during CpG-ODN stimulated DC vaccination, because CpG-ODN stimulation makes DCs switch their antigen processing pathway toward the MHC class I type than the class II type, resulting in a dramatic induction of IFN-γ secreting CD8^+^ T cell responses [29]. These IFN-γ secreting CD8^+^ T cells are cytotoxic T lymphocytes in nature [30]. Our results showed that there was a significant enhancement of splenic IFN-γ^+^ CD8^+^ T-cells when parasitized mice were vaccinated with SLA-CpG-DCs compared to infected or unprotected group of parasitized mice (Figure 4A). To establish the role of CXCL10 in the induction of IFN-γ^+^ CD8^+^ T cells following SLA-CpG-DCs vaccination we depleted CXCL10 in vivo during the course of the vaccination. We observed significant reduction of IFN-γ^+^ CD8^+^ T cells in mice depleted of CXCL10 in contrast to non-depleted controls suggesting a direct role of CXCL10 in the generation of IFN-γ^+^ CD8^+^ T cells in the SLA-CpG-DCs vaccinated mice.

To determine whether SLA-CpG-DCs vaccination generated CD8^+^ T cells can direct antigen-specific immune responses against *L. donovani*, antigen-specific proliferative responses were measured using purified CD8^+^ T cells from different groups of vaccinated mice. The CD8^+^ population purity was routinely confirmed to be around 98% (Figure S1).Significantly higher proliferation of CD8^+^ was observed in the CD8^+^ cells isolated from the SLA-CpG-DCs vaccinated mice compared to CD8^+^ T cells from only infected, only CpG-DCs or SLA-control-ODN-DC vaccinated sets (Figure 4B). In a separate study, we have evaluated CD8^+^ T-cell dependent production of IFN-γ by stimulating purified CD8^+^ T cells from differently vaccinated groups, *in vitro* with SLA for 72 hours. Significantly higher IFN-γ production was observed in CD8^+^ T cells isolated from SLA-CpG-DC treated parasitized mice, as compared to CD8^+^ T cells from only infected or unprotected group of mice (Figure 4C). These data indicate that SLA-CpG-DCs have a significant impact on the generation of an antigen-specific CD8^+^ T cell response against the parasite.

To assess the contribution of CD8^+^ T cells in the induction of protective immunity after DC-mediated vaccination in vivo, BALB/c mice were transiently depleted of CD8^+^ T cells by intra-peritoneal injection of CD8 depleting antibody. While animals that had been immunized with SLA-CpG-DCs were fully protected against the infection, CD8 depleted SLA-CpG-DCs vaccinated mice showed almost no protection signifying the role of CD8^+^ T cells in the protective immunity following DC vaccination (Figure 4D). Besides, SLA-CpG-DCs vaccinated mice receiving control antibody show full protection against *Leishmania* infection (Figure 4D).

### 4. CXCL10 Depletion Reduces the Cytotoxicity of CD8^+^ T Cells in SLA-CpG-DCs Vaccinated Parasitized Mice

**Figure 5:**
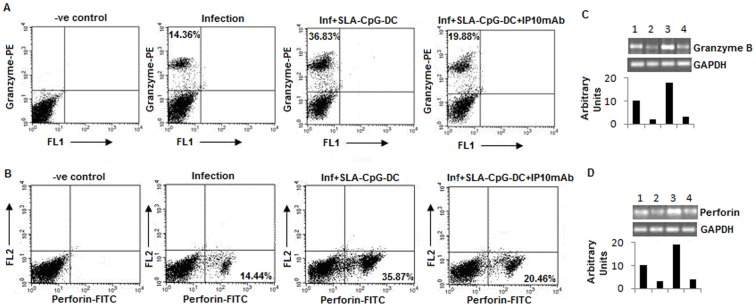
CXCL10 depletion reduces the cytotoxicity of CD8^+^ T cells in SLA-CpG-DCs vaccinated parasitized mice. (**A, B**) CD8^+^ T cells (1×10^6^) were purified from spleens of infected, SLA-CpG-DCs vaccinated and CXCL10 depleted vaccinated mice (methods) after 4 weeks post infection, harvested and stimulated for 4 h with SLA (10 µg/ml), followed by fixation and staining for Granzyme B-PE (**A**) and Perforin-FITC (**B**) as mentioned in materials and methods. The data was analyzed by flow cytometry. These data were from one of three experiments conducted in the same way with similar results. (**C, D**) A separate set of CD8^+^ T cells (2×10^6^), of the above mentioned groups was collected in Trizol for mRNA extraction and subjected to RT (Reverse Transcriptase) PCR as described in Methods. Expression of granzyme B (**C**) and perforin (**D**) from CD8^+^ T cells was observed in upper panel; Group 1: Control, Group 2: Infection, Group 3: Infection+ SLA-CpG-DCs and Group 4: Infection+ SLA-CpG-DCs and Group+ CXCL10 mAbs. Lower panel shows GAPDH expression levels. Band intensities were analyzed by densitometry. Results are representative of three experiments conducted in the same way with similar results.

The cytotoxicity of CD8^+^ T cells is exerted through two processes: one is mediated through perforin, a membrane pore forming molecule, and granzyme B which activate caspase dependant apoptosis of the target cells [31]. Our data revealed that there was an increase in both perforin^+^ and granzyme B^+^ CD8^+^ T cells after SLA-CpG-DCs vaccination compared to infected group of mice (Figure 5A–B). Depletion of CXCL10 reduces both perforin^+^ and granzyme B^+^ CD8^+^ T following SLA-CpG-DCs vaccination indicating a direct role of CXCL10 in perforin and granzyme B^+^ activation in CD8^+^ T cells of the vaccinated mice. We also checked mRNA levels of both perforin and granzyme B in purified CD8^+^ T cells, which was also found to be increase in the SLA-CpG-DCs vaccinated parasitized mice compared to infected mice. The mRNAs of both perforin and granzyme B was also reduced following CXCL10 depletion (Figure 5C–D). Therefore, these results indicated that CXCL10 contributed towards the generation of perforin^+^ granzyme B^+^ CD8^+^ T cells following SLA-CpG-DCs vaccination.

**Figure 6:**
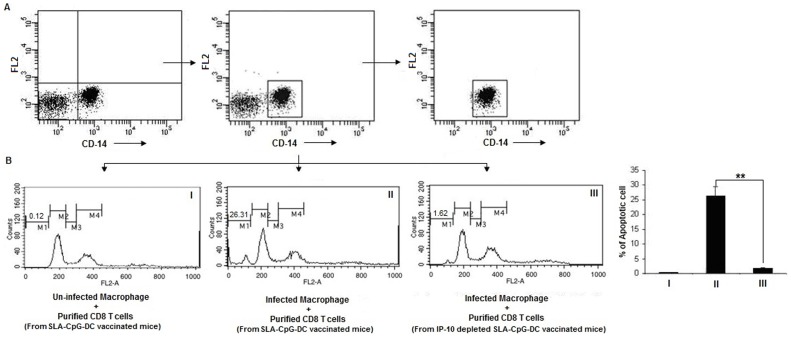
CD8^+^ T cell cytotoxicity. CD8^+^ T cells purified from SLA-CpG-DCs vaccinated parasitized mice 28 days after infection were co-incubated with autologous uninfected (I) and *Leishmania*-infected macrophages (II) in a 10∶1 ratio. In another set, CD8^+^ T cells were purified from CXCL10 depleted SLA-CpG-DCs vaccinated parasitized mice 28 days after infection and co-incubated with autologous *Leishmania*-infected macrophages in a 10∶1 ratio (**III**). After 4 h, the cells were harvested and stained with anti-CD14-FITC antibodies to select the target macrophages (**A**). The dot plots were derived from the gated events based on the region encircling positive cells. This CD14-FITC positive population were analyzed for propidium iodide staining to detect killed macrophages (**B**). M1: Apoptotic peak, M2: G1 peak, M3: S peak and M4: G2+M peak. The data was analyzed on a flow cytometer (FACS Calibur), using the Cell Quest program. These data were from one of three experiments conducted in the same way with similar results. The error bars represent mean ± SD. **P<.001, compared to SLA-CpG-DC vaccinated parasitized mice.

Antigen-primed CD8^+^ T cells have the capacity to destroy autologous infected macrophages [32]. To assess the cytotoxicity of these primed CD8^+^ T cells purified CD8^+^ T cells were isolated from SLA-CpG-DCs vaccinated and CXCL10 depleted SLA-CpG-DCs vaccinated *L. donovani* infected mice 28 days following infection and were co-cultured with autologous *L.donovani*-infected macrophages in a 10∶1 ratio. After 4 h, the co-cultured cells were harvested and stained with anti-CD14-FITC antibodies to select the target macrophages. They were also analyzed by propidium iodide staining to detect the killed macrophages. Our data revealed that macrophages co-cultured with CD8^+^ T cells from SLA-CpG-DCs vaccinated mice showed a significant higher degree of killing than macrophages co-cultured with CD8^+^ T cells from CXCL10 depleted SLA-CpG-DCs vaccinated mice (Figure 6). In addition, there is no effect of these primed CD8^+^ T cells from SLA-CpG-DCs vaccinated parasitized mice on uninfected macrophages signifying antigen-dependant specific killing (Figure 6).

### 5. Humoral Responses in SLA-CpG-DCs Vaccinated *L. donovani*-infected Mice

**Figure 7:**
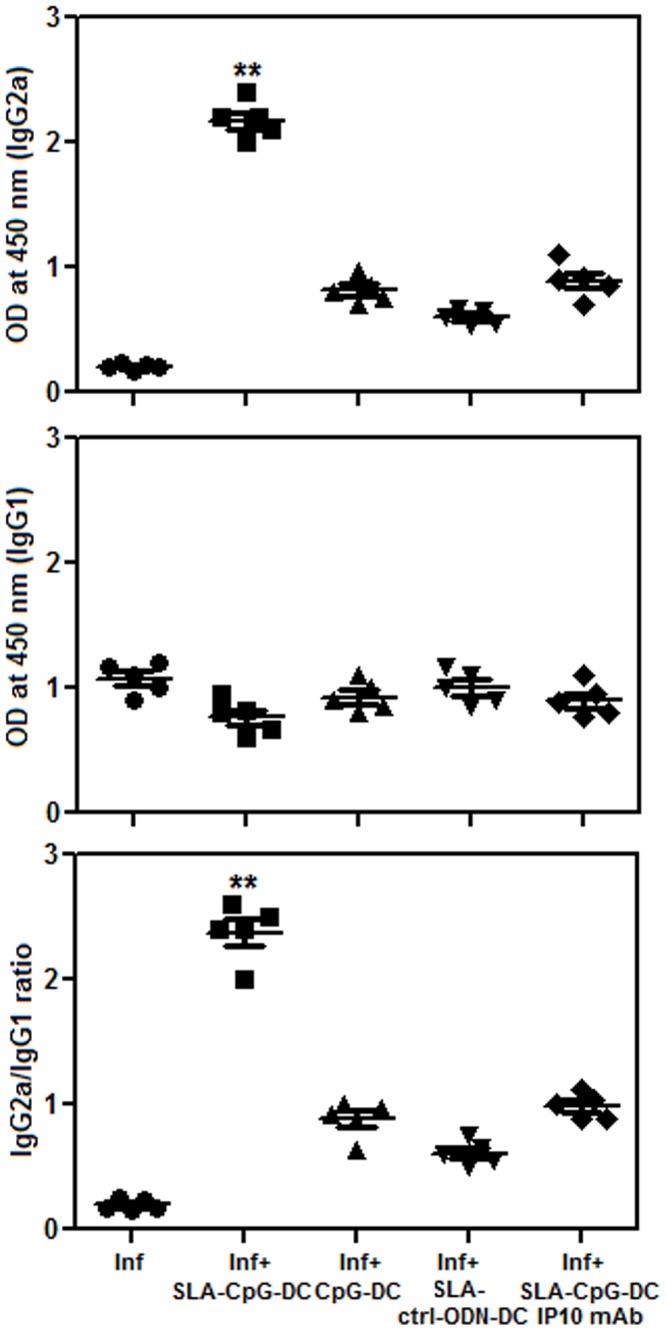
SLA-CpG-DCs vaccination induces enhanced production of IgG2a. Leishmania specific Ab responses in infected and differently vaccinated *L. donovani*-infected BALB/c mice were studied. Sera from infected and differently vaccinated BALB/c mice were collected at 28 Days and analyzed for Leishmania Ag-specific anti-IgG1 and anti-IgG2a levels by ELISA. Results are from 3 independent experiments and represent the mean values ± standard errors of the means for 5 animals per group. **P<.001, compared to infected mice.

To determine the type of humoral immune response induced by SLA-CpG-DCs against experimental VL, four weeks after the infection, IgG1 and IgG2a responses in the sera of different groups of mice was also determined. IgG1 is induced by IL-4, a Th2 cytokine, whereas IgG2a is induced by IFN-γ, a Th1 cytokine [33]. Serum from SLA-CpG-DCs vaccinated mice mounted 10.3 fold higher SLA specific IgG2a compared to only infected mice, and almost 1.38 fold decrease of SLA specific IgG1 compared to only infected mice (Figure 7). This protected group of mice contained low levels of *L.donovani*-specific IgG1 and high levels of parasite-specific IgG2a Ab and exhibited the highest IgG2a/IgG1 ratio (2.38) (Figure 7). This indicates that vaccination with SLA-CpG-DCs induced a shift toward a Th1-dominated immune response after *L. donovani* infection.

## Discussion

The induction of an effective cellular immune response against *Leishmania* pathogen requires a strong clonal expansion of antigen-specific CD8^+^ T-cells [34], [35]. *L. donovani* evades this defence by restraining the effector functions of T-cell responses [36]. In this study, we demonstrated that DCs activated with a prominent TLR ligand CpG-ODN and pulsed with SLA induce a highly effective protective immunity against leishmanial pathogenesis via generation of antigen-specific CD8^+^ T-cells *in vivo*. Previously it was reported that the protection conferred by antigen pulsed CpG-ODN stimulated DCs is independent of DC-derived IL-12 [4]. But there is no clear understanding about the role played by CpG-ODN stimulated DCs in the generation of antigen-specific CD8^+^ T-cells in the context of leishmanial pathogenesis. Here, we demonstrate for the first time that the induction of a CD8^+^ T cell mediated anti-leishmanial protective immunity by SLA-CpG-DCs is entirely dependent on the production of CXC chemokine CXCL10 by these DCs. Our *in vivo* experiments demonstrated that SLA-CpG-DC vaccination could significantly restrict parasite growth in spleen and liver after 28 days of infection (Figure 2 A–B), and this protective immunity is abrogated with anti- CXCL10 mAbs resulting in the reappearance of high hepatic and splenic parasitic growth in the immunized animals (Figure 3).

The type of DC stimulus is a critical factor which determines the capacity of DCs to direct a T cell response. TLR ligand stimulated DCs are capable of cross-presenting exogenous antigens to CD8^+^ T-cells by attracting naïve cytotoxic T lymphocytes though the generation of a chemokine gradient [37], [38]. Our *in vitro* experiments showed that stimulation of DCs with CpG-ODNs resulted in the secretion of CXCL10, a CXCR3 ligand, which might attract naïve CTLs and increase the likelihood of the encounter of CTL’s with licensed DCs for effective cross priming. The effector function of CD8^+^ T cells relies on CXCR3 signaling. CXCR3 signaling is critical for the chemotaxis, activation, and cytotoxic responses elicited by CD8^+^ T cells [25]. In agreement with the above findings, we observed an enhancement of IFN-γ secreting leishmanial antigen specific CD8^+^ cytotoxic T lymphocytes upon SLA-CpG-DC vaccination (Figure 4A). Depletion of CXCL10 efficiently inhibited both the generation and antigen dependant proliferation of these CD8^+^ cytotoxic T lymphocytes (Figure 4 A, B).

Previous studies have demonstrated that CD8^+^ effector responses are required for protection against *Leishmania* infection [34], [35], [39], [40]. Recently, it has been shown that, adoptive transfer of CD8^+^ T cells reduced *L. donovani* infection in susceptible BALB/c mice by eliminating CD4^+^CD25^+^ T regulatory cells via the induction of cytotoxic mediators, granzyme and perforin [41]. Our data revealed that there was a significant increase in granzyme and perforin secreting CD8^+^ T cells which most likely contributes to the protection conferred by the SLA-CpG-DCs (Figure 5 A, B) as depleting these CD8^+^ T cells results in significant increase in the liver and spleen parasitic burden (Figure 4D).

CXCL10 plays a critical role in this activation of granzyme and perforin secreting CD8^+^ T cells because mice lacking CXCL10 receptor showed reduced CTL activity and impairment of perforin, and granzyme B expression by CD8^+^ T cells [25]. Neutralization of CXCL10 in the immunized group reduces both granzyme and perforin secreting CD8^+^ T cells as well as their cytotoxic properties (Figure 5 A–B, 6). CXCL10, with known anti-leishmanial property can induce host-protective immune response by regulating the CD4^+^CD25^+^ T regulatory cell functioning in *Leishmania donovani*-infected mice [22], [42], [43]. Thus the changes in effector CD8^+^ T cell functioning following SLA-CpG-DCs vaccination can be further correlated with T regulatory cell functioning during leishmanial pathogenesis as CXCL10 can regulate the balance between effector T cells and Treg cells. Therefore, these findings demonstrate a critical role of CXCL10 in effector T cell activation in this infectious disease model following SLA-CpG-DCs vaccination.

Collectively, these findings illustrate that upon SLA-CpG-ODN stimulation DC might function as a potent adjuvant for induction of an antigen-specific protective cellular immunity against leishmania-induced pathogenesis. The evidence presented here indicates that the anti-leishmanial protective immunity by SLA-CpG-DC vaccination is dependent on the activation CXC chemokine CXCL10. Inhibition of CXCL10 resulted in abrogation of the vaccination effect as indicated by a failure of CXCL10 -depleted mice to control a subsequent leishmanial challenge. The protection also requires the activation of effector CD8^+^ T cells. These findings were supported by a decrease in CD8^+^ T cell activation in CXCL10 -depleted mice associated with the reduction of perforin, granzyme B secretion and decrease in the number of Ag-specific CD8^+^ T cells producing IFN-γ. Taken together, these data describe a novel role for CXCL10 in the generation of protective immunity induced by SLA-CpG-DC vaccination, representing an immune response that leads to disease control against experimental VL.

## Supporting Information

Figure S1 **Purity of CD8^+^ T cells.** Spenocytes (1×10^6^) were isolated from spleen of differently vaccinated mice 28 days after infection. Splenocytes were then stained with CD8-FITC antibody and analyzed on a flow cytometer. The purity of these CD8^+^ T cells was found to be around 98%.
(TIF)
