# Supplementary figures and images for: CXCL10 Is Critical for the Generation of Protective CD8 T Cell Response Induced by Antigen Pulsed CpG-ODN Activated Dendritic Cells
Source: PLoS One. 2012 Nov 7;7(11):e48727. doi: 10.1371/journal.pone.0048727 (PMC3492407; doi:10.1371/journal.pone.0048727)

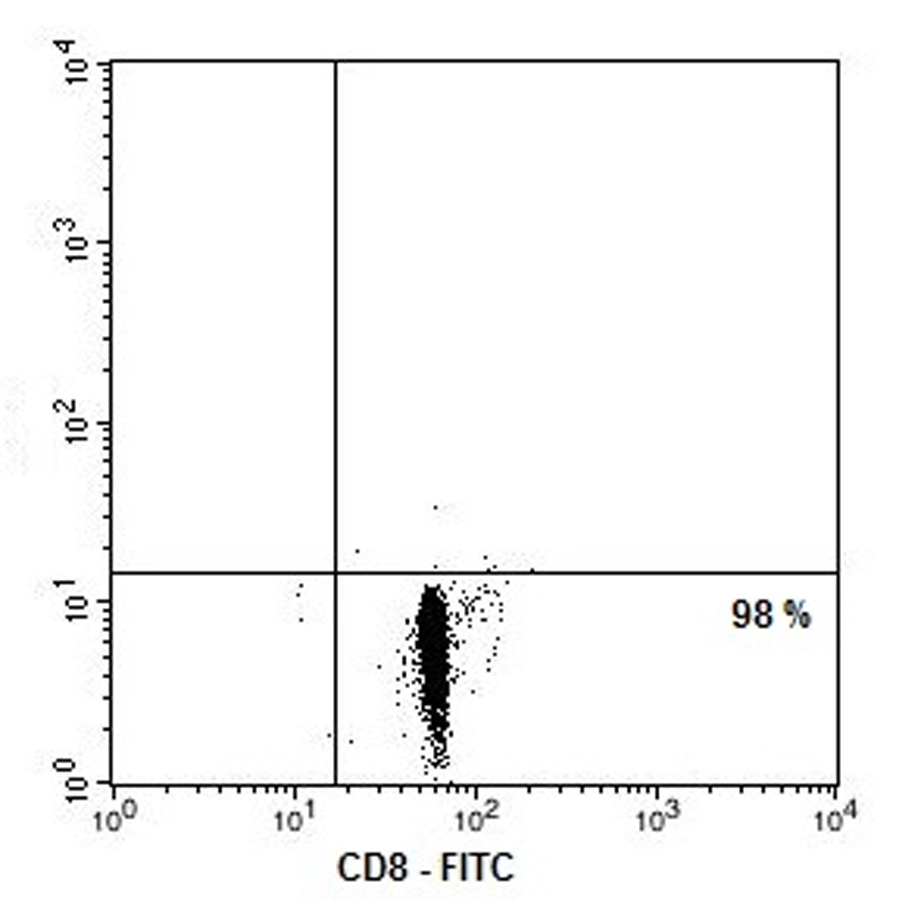

Supplement: Figure S1 — Purity of CD8+ T cells. Spenocytes (1×10^6^) were isolated from spleen of differently vaccinated mice 28 days after infection. Splenocytes were then stained with CD8-FITC antibody and analyzed on a flow cytometer. The purity of these CD8+ T cells was found to be around 98%. (TIF) [file pone.0048727.s001.tif]
